# Definitive Characterization of CA 19-9 in Resectable Pancreatic Cancer Using a Reference Set of Serum and Plasma Specimens

**DOI:** 10.1371/journal.pone.0139049

**Published:** 2015-10-02

**Authors:** Brian B. Haab, Ying Huang, Seetharaman Balasenthil, Katie Partyka, Huiyuan Tang, Michelle Anderson, Peter Allen, Aaron Sasson, Herbert Zeh, Karen Kaul, Doron Kletter, Shaokui Ge, Marshall Bern, Richard Kwon, Ivan Blasutig, Sudhir Srivastava, Marsha L. Frazier, Subrata Sen, Michael A. Hollingsworth, Jo Ann Rinaudo, Ann M. Killary, Randall E. Brand

**Affiliations:** 1 Van Andel Research Institute, Grand Rapids, MI, United States of America; 2 Fred Hutchinson Cancer Research Center, Seattle, WA, United States of America; 3 The University of Texas MD Anderson Cancer Center, Houston, TX, United States of America; 4 University of Michigan, Ann Arbor, MI, United States of America; 5 Memorial Sloan Kettering Cancer Center, New York, NY, United States of America; 6 University of Nebraska Medical Center, Omaha, NE, United States of America; 7 University of Pittsburgh Medical Center, Pittsburgh, PA, United States of America; 8 Northshore University Healthsystems, Evanston, IL, United States of America; 9 Palo Alto Research Center, Palo Alto, CA, United States of America; 10 University Health Network, Toronto, ON, Canada; 11 National Cancer Institute, Rockville, MD, United States of America; University of Szeged, HUNGARY

## Abstract

The validation of candidate biomarkers often is hampered by the lack of a reliable means of assessing and comparing performance. We present here a reference set of serum and plasma samples to facilitate the validation of biomarkers for resectable pancreatic cancer. The reference set includes a large cohort of stage I-II pancreatic cancer patients, recruited from 5 different institutions, and relevant control groups. We characterized the performance of the current best serological biomarker for pancreatic cancer, CA 19–9, using plasma samples from the reference set to provide a benchmark for future biomarker studies and to further our knowledge of CA 19–9 in early-stage pancreatic cancer and the control groups. CA 19–9 distinguished pancreatic cancers from the healthy and chronic pancreatitis groups with an average sensitivity and specificity of 70–74%, similar to previous studies using all stages of pancreatic cancer. Chronic pancreatitis patients did not show CA 19–9 elevations, but patients with benign biliary obstruction had elevations nearly as high as the cancer patients. We gained additional information about the biomarker by comparing two distinct assays. The two CA 9–9 assays agreed well in overall performance but diverged in measurements of individual samples, potentially due to subtle differences in antibody specificity as revealed by glycan array analysis. Thus, the reference set promises be a valuable resource for biomarker validation and comparison, and the CA 19–9 data presented here will be useful for benchmarking and for exploring relationships to CA 19–9.

## Introduction

Advances in knowledge about pancreatic cancer have generated enthusiasm about the prospects for significant progress against this deadly disease [1, 2]. Among other areas, the field has witnessed advances in understanding the genetic initiation and progression of the disease [3], the role of the stroma in promoting cancer and in obstructing systemic therapies [4–7], and the role of plasticity in the cancer cell [8]. While this information will be foundational in the development of effective therapies, improvements in survival also will depend on better detection, diagnostics, and treatment decisions based on individual patient characteristics. We need to advance our ability to detect a pancreatic cancer at an early stage, when it is potentially curable by surgical resection, and we need better ways to determine the route of care that will be most effective for each patient.

Molecular biomarkers promise to provide such precise, patient-specific information [9], but their development and implementation is challenging and slow. In pancreatic cancer research, a major bottleneck for the development of molecular biomarkers is the limited resources for assessing and comparing candidate biomarkers using samples collected at early stages of cancer development, when resection for cure is possible. A significant amount of time usually is required before an accurate assessment of a biomarker is possible and before decisions about further investment can be made. More often than not, promising results in early studies are not substantiated in follow up studies, or the performance of a biomarker is not consistent between studies [10, 11]. A consistent and systematic approach to evaluating candidate biomarkers is required.

To address the need for the evaluation of pancreatic cancer biomarkers, a collaborative group within the Early Detection Research Network (EDRN) recently developed a reference set of human specimens. The motivation for developing the reference set was to enable definitive evaluation of candidate biomarkers for pancreatic cancer, to provide accurate comparisons between candidate biomarkers, and to test the combined use of disparate biomarkers. A common set of specimens, collected under rigorous standards at multiple institutions and comprising the patient populations most relevant to the clinical requirements, is necessary for achieving these goals [12]. Several principles guided the creation of the set. The set was to include samples from multiple institutions, so that it is not representative of only one geographical area; sample collection was to follow a single, detailed standard operating procedure, to prevent the introduction of bias into the set; the patients were to include many with resectable cancer, which is the most difficult to detect yet the most important for potential positive impact; and the control subjects were to include both healthy people and patients with benign conditions of the pancreas, because certain benign conditions can be hard to distinguish from pancreatic cancer and could cause elevations in cancer biomarkers.

Regarding the patient population, we chose to assemble samples from patients with stage I or II cancer as confirmed by surgical pathology. Such patients are eligible for surgical treatment and on average have significantly better outcomes than the rest of pancreatic cancer patients. Detecting cancer even earlier, i.e. at carcinoma in-situ or PanIN–3, would be preferable to detection at stage I because patients with stage I typically still develop recurrence after surgery. At present we do not have a way to routinely confirm the presence of PanIN–3, so assembling samples from a cohort of such patients is not yet possible. An alternate approach used previously was to assemble samples that had been collected prior to the diagnosis of pancreatic cancer, identified through the examination of follow-up information from massive public-health studies to find subjects who eventually developed pancreatic cancer [13, 14]. Such samples are precious for exploring the feasibility of screening for cancer, but they are not designed to test for detection prior to stage I or II because the stage of disease is not known. In addition, because of the difficulty in obtaining such samples, they normally are persevered for only a small number of studies. Therefore we pursued the assembly of a sample set that could be used for many studies and that would be relevant to an important goal in pancreatic cancer treatment, the detection of more cancers at a stage that is eligible for surgery.

The CA 19–9 assay is the current best serological biomarker for pancreatic cancer. The CA 19–9 monoclonal antibody was raised against a colorectal cancer cell line [15], and the antigen it binds is a carbohydrate structure attached to a variety of proteins and lipids [16]. Although its blood levels are strongly associated with pancreatic cancer—it is elevated in 70–80% of pancreatic cancer patients and in about 20% of patients with benign conditions of the pancreas [17]—its performance is not sufficient for diagnosing cancer, as the risk of both false negative and false positive diagnoses is unacceptably high. About 5% of pancreatic cancer patients do not elevate CA 19–9 due to germline mutations resulting in inability to produce the glycan [18], and another group of patients do not elevate CA 19–9 for unknown reasons. The non-specific elevations in CA 19–9 result mainly from damage to the bile duct or portions of the pancreatic ducts that are coated with the CA 19–9 antigen. New biomarkers for the diagnosis of resectable cancer must perform better than CA 19–9, both in sensitivity and specificity, either as a single biomarker or more likely in combination.

The goals of the present study were to 1) determine the performance of CA 19–9 in the reference set; 2) more clearly define the performance of CA 19–9 for stage I-II disease and in reference to the potentially confounding conditions of benign biliary obstruction and chronic pancreatitis; and 3) compare the performance and define the origins of differences between distinct CA 19–9 assays. The first goal was necessary to give a benchmark by which we can compare and evaluate candidate biomarkers. The second goal was necessary because many of the previous studies of CA 19–9 were heavily weighted toward late-stage cancer, mainly because samples from late-stage cancer patients are more readily available. Patients with stage I-II disease have the potential for improved outcomes through surgery, so the accurate and early detection of this level of disease is critically important. The third goal was necessary because the previous studies of CA 19–9 in pancreatic cancer have divergent results, likely owing to differences in the specificities of the antibodies [19] or differences in the assay platforms. It is important to understand the implications of these differences for decisions for individual patients. In addition, more information about the glycans bound by each CA 19–9 antibody potentially would help to characterize the glycans produced by cancer patients and to formulate strategies for detecting a greater percentage of patients than possible now.

## Materials and Methods

### Eligibility criteria

Each site conducted sample collection using a protocol and written consent form that were approval by their Institutional Review Board. Subjects were required to provide written, informed consent. The sites participating in the sample collection were the University of Pittsburgh Medical Center, Memorial Sloan Kettering Cancer Center, the University of Michigan, the University of Nebraska, and Northshore University Healthsystem (previously known as Evanston Northwestern Healthcare). The following inclusion criteria were required for pancreatic cancer cases and controls. The control groups included chronic pancreatitis patients, acute benign biliary obstruction patients, and healthy subjects.

#### Pancreatic cancer cases

1. Subjects underwent curative pancreatic resection for an adenocarcinoma including negative margins and preferably Stage 1 or 2A (absence of lymph node metastases).
2. No prior history of any other malignancy except nonmelanoma skin cancers for ten years.

#### Chronic pancreatitis cases

1. All subjects must have had at least two of the radiological criteria listed below, unless a subject had a history of pancreatic exocrine insufficiency, in which case only one radiological criterion was required.
  - Abdominal ultrasound that is consistent with chronic pancreatitis by standard radiological criteria
  - Abdominal CT scan consistent with chronic pancreatitis by standard radiological criteria (i.e., calcifications, dilated pancreatic duct, irregular contour of the gland, cystic lesions).
  - Endoscopic retrograde cholangiopancreatography (ERCP) exam consistent with chronic pancreatitis by standard radiological criteria (dilated tortuous main pancreatic duct with irregular secondary branches, intraductal calculi).
  - Endoscopic ultrasound consistent with chronic pancreatitis by standard sonographic criteria.
  - Pancreatic calcifications identified on plain film of the abdomen.
2. All subjects had an imaging study of the pancreas within 3 months of study enrollment which did not suggest a pancreatic mass.
3. All subjects had a stable clinical history over the past year with no suspicion for cancer (weight loss, jaundice, or change in abdominal symptoms).
4. All subjects had no prior history of any other malignancy except non-melanoma skin cancers for the past ten years.
5. All subjects had no family history of pancreatic cancer.

#### Acute benign biliary obstruction cases

1. All subjects met all of the following clinical criteria:
  - Elevation of serum bilirubin level greater than 2.0 mg/dL
  - Dilated extrahepatic biliary systems demonstrated on imaging study
  - Blood sample obtained prior to any corrective intervention.
2. All subjects had biliary obstruction that was of benign etiology such as common bile duct stone or benign biliary stricture.
3. Patients with primary sclerosing cholangitis (PSC) were excluded.
4. All subjects had a complete imaging study performed of the pancreas that did not suggest a pancreatic cancer, such as a discrete mass lesion.
5. All subjects had no prior history of any other malignancy except non melanoma skin cancers for the past ten years.
6. All subjects had no family history of pancreatic cancer.

#### Healthy controls

All subjects met the following criteria.

1. Age, race and sex matched to qualified pancreatic cancer cases.
2. No family history of pancreatic cancer.
3. No personal history of acute pancreatitis or biliary obstruction as defined above.
4. No concurrent abdominal pain.
5. No concurrent unexplained weight loss.
6. No prior history of any other malignancy except non-melanoma skin cancers for the past ten years.

There was no extended follow up beyond the time that the samples were sent at the end of the collection period. While the possibility exists that one or more of the controls had incipient cancer at the time of sample collection, the effect on the study likely would be negligible. Due to how rare the disease is in the general population with a life-time risk of 1 in 71 patients, it would only be anticipated that at most one of these patients at a general population risk for PC will ever develop a cancer in their life-time.

### Serum and plasma collection

All collections took place following informed consent of the participants and prior to any surgeries or procedures. The samples were collected from February 21, 2005 to April 20, 2011, and the experiments were performed in 2013 and 2014. All blood samples were collected according to the EDRN standard operating procedure. All samples were frozen at -70°C or colder within 4 hours of time of collection. Four mL of serum and 2 mL of plasma (using EDTA as the anticoagulant) were shipped from participating sites to NCI Biorepository in Frederick, Maryland. The specimens were transferred to the repository in a manner that prevented thawing using approved transporter techniques. Aliquots were sent on dry ice to the sites performing the CA 19–9 analyses, and no aliquots were thawed more than three times total prior to use.

The healthy controls were collected from 4 out of the 5 sites with contributions of the controls matching the overall contributions from each site (Table 1). We monitored the control recruitment to ensure there was reasonable matching of the controls to the cases in age, race, and sex, but we relaxed the algorithm somewhat with the chronic pancreatitis patients owing to the earlier age of onset relative to pancreatic cancer.

**Table 1:** Characteristics of study subjects.

	Healthy control(N = 61)		Chronic pancreatitis(N = 62)		Acute benign biliary obstruction(N = 31)		Pancreatic cancer (N = 98)		Total (N = 252)	
	N	%	N	%	N	%	N	%	N	%
Centers										
ENH	0	(0%)	0	(0%)	0	(0%)	15	(15%)	15	(6%)
MSKCC	9	(15%)	0	(0%)	0	(0%)	24	(25%)	33	(13%)
Pitt	26	(43%)	36	(58%)	14	(45%)	26	(27%)	102	(40%)
UMHS	12	(20%)	22	(35%)	16	(52%)	19	(19%)	69	(27%)
UNMC	14	(23%)	4	(6%)	1	(3%)	14	(14%)	33	(14%)
Ages										
<50	1	(2%)	7	(11%)	3	(10%)	1	(1%)	12	(5%)
50~59	19	(31%)	24	(39%)	6	(19%)	13	(13%)	62	(25%)
60~69	25	(41%)	17	(27%)	10	(32%)	35	(36%)	87	(35%)
70~79	10	(16%)	12	(19%)	7	(23%)	29	(39%)	58	(23%)
>80	6	(10%)	2	(3%)	5	(16%)	20	(21%)	33	(13%)
Gender										
Male	25	(41%)	36	(56%)	12	(39%)	43	(44%)	116	(46%)
Female	36	(59%)	26	(44%)	19	(61%)	55	(56%)	136	(54%)
Race										
White	55	(90%)	57	(92%)	29	(94%)	89	(92%)	230	(91%)
Asian	0	(0%)	0	(0%)	0	(0%)	3	(3%)	3	(1%)
African-American	6	(10%)	4	(6%)	2	(6%)	1	(1%)	13	(5%)
Native and others	0	(0%)	1	(2%)	0	(0%)	5	(4%)	6	(2%)
Tumor Stages										
IA							7	(7%)		
IB							8	(8%)		
II							1	(1%)		
IIA							40	(41%)		
IIB							42	(43%)		

ENH, Evanston Northwestern Healthcare; MSKCC, Memorial Sloan Kettering Cancer Center; Pitt, University of Pittsburgh Medical Center; UMHS, University of Michigan Health Services; UNMC, University of Nebraska Medical Center.

### Definitions of tumor stages

The pancreatic adenocarcinomas were staged according to the criteria in the American Joint Committee on Cancer (AJCC) Staging Manual 7^th^ edition. The following definitions of tumor extent and nodal status were used: T1 = Tumor limited to the pancreas ≤2 cm in greatest dimension; T2 = Tumor limited to the pancreas >2 cm in greatest dimension; T3 = Tumor extends beyond the pancreas but without involvement of the celiac axis or the superior mesenteric artery; N0 = No regional lymph node metastasis; and N1 = Regional lymph node metastasis. The stages were defined as: Stage 1a = T1N0M0; Stage 1b = T2N0M0; Stage 2a = T3N0M0; and Stage 2b = T1-3N1M0.

### CA 19–9 assays

Two laboratories ran CA 19–9 assays on aliquots of the plasma samples that were received without any identifying information. One assay, the EIA–1474 kit [Lot #RN–45868] from DRG International (Springfield, NJ), was run in the laboratory of Dr. Killary according to previously published methods [20]. The other assay was developed and run in the laboratory of Dr. Haab using a previously published protocol [19, 21, 22]. In the subsequent text, we refer to the former as “Assay 1” and the latter as “Assay 2.” The DRG kit nominally is approved only for serum, but it previously was used for plasma to achieve results similar to those achieved using serum [20]. In this study, we further validated its use with plasma by confirming statistical equivalence with Assay 1 and with the Abbott Architect platform (see Results). A subset of the samples (n = 82) was analyzed using the CA 19–9 assay on the Abbott Architect Immunoassay platform at the University Health Network in Toronto, Canada. See the S1 File for additional information about the protocols and assay characteristics.

### Statistical analysis

For each group, we compared the CA19-9 distributions between the two CA19-9 assays using the paired t-test (on log-transformed CA19-9) and the Wilcoxon Signed Rank test. The Spearman rank correlation coefficient between the two assays was also computed. For descriptive analyses, we generated a boxplot for each CA19-9 assay and each group; the geometric means of individual assays and their 95% Wald confidence intervals also were computed.

For pairwise comparisons among the healthy, benign biliary obstruction, chronic pancreatitis, and cancer groups, we performed two-sample t-tests (on the log-transformed CA19-9 values) and the Wilcoxon Rank Sum tests. In addition, for each paired control and case group, we generated a nonparametric estimate of the receiving operating characteristics (ROC) curve [23] and the area-under-the-curve (AUC) for the individual CA19-9 assays and for a linear combination of the log-transformed values from the two assays derived from logistic regression models. The bootstrap procedure with 500-fold resampling was used for constructing the confidence intervals of the AUCs of the individual assays, of the differences in AUC between the two CA19-9 assays, and of the difference in AUC between the individual CA19-9 assays and the combined assays.

For the individual CA19-9 assays, we examined the sensitivities and specificities using the clinical cutoff of 37 U/mL as well as a derived cutoff that maximizes the sum of sensitivity and specificity. We also estimated sensitivity and specificity based on combinations of the two CA19-9 assays using AND/OR rules. The bootstrap procedure with 500-fold resampling was used for constructing the confidence intervals for sensitivity, specificity, and the sum of sensitivity and specificity.

### Glycan array experiments and analysis

The core laboratory for Glycan Array Synthesis (part of the Consortium for Functional Glycomics, CFG) at Emory University performed the glycan array experiments and primary analyses. The array version used here was 5.1, containing 610 unique glycans. The experiments followed published protocols [24]. We used the program GlycoSearch [25] to analyze the glycan array data.

## Results

### CA 19–9 in the reference set

The reference set included serum and plasma samples collected at five different institutions under a common standard operating procedure. The samples are from 98 patients with stage I-II pancreatic cancer, 62 patients with chronic pancreatitis, 31 patients with benign biliary obstruction, and 61 healthy control subjects (Table 1).

We determined the CA 19–9 values in the plasma samples using two different assays, one a commercially-available kit (referred to as Assay 1), and the other an in-house system (referred to as Assay 2). We used two different assays in order to account for potential differences between assays, given previous observations of such differences [26, 27]. We tested the reliability the assays used here by comparisons with an automated platform (Abbott Architect CA 19–9 Immunoassay) for 82 of the samples distributed across the patient groups. The values within each of the patient groups were statistically equivalent between all three assays, except for slightly higher levels among healthy controls for Assay 2 relative to the Abbott assay (Table A1 in S3 File), and the discrimination of cancer from the control groups was statistically equivalent between all assays (Table B in S3 File and S1 File). This result confirms the reliability of the results obtained using Assays 1 and 2 and their general equivalence with automated platforms.

**Fig 1:**
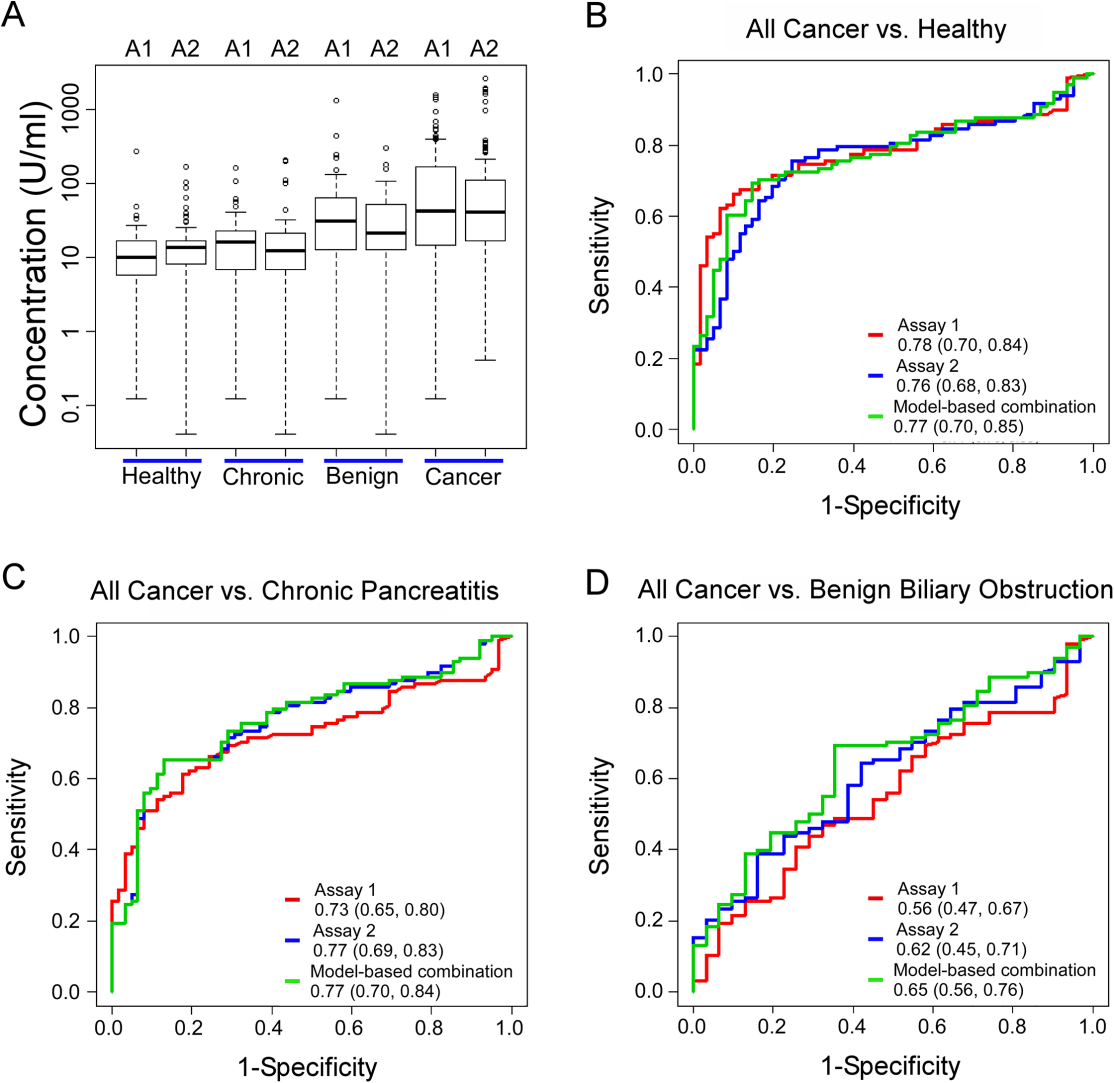
Differentiating pancreatic cancer from control subjects by two different CA 19–9 assays. A) CA 19–9 levels in each group. We present the log-transformed values to better visualize all ranges of the values. The boxes indicate the quartiles of the distributions, the horizontal lines in the boxes indicate the medians, and the dashed lines give the ranges, with individual outliers indicated by the circles. A1, Assay 1; A2, Assay 2. B-D) Receiver-operator-characteristic (ROC) curves comparing all pancreatic cancer patients to the indicated control groups. The legends specify the area-under-the-curves (AUCs) for each assay, with the ranges of the 95% confidence intervals.

We first examined the distributions of the values for each of the assays in the various patient groups (Fig 1A). The healthy subjects and chronic pancreatitis patients had the lowest levels; benign biliary obstruction patients had significantly higher levels (p-value is less than 0.0001 and equal to 0.006 by Wilcoxon Rank Sum test, relative to healthy subjects for Assay 1 and 2 respectively); and cancer patients had the highest levels (p<0.0001 relative to healthy subjects based on either t-test or Wilcoxon Rank Sum test for each assay). The two assays showed equivalent trends. Detailed results about the geometric means of the individual assays and their 95% confidence intervals are presented in Table C in S3 File, and the p-values for the comparisons between patient groups are presented in Table D in S3 File.

We asked whether the CA 19–9 levels were different between the earlier-stage (stages Ia, Ib, and IIa) and the later-stage (stage IIb) patients. The CA19-9 levels were not significantly different between the two groups (p-values based on t-test and Wilcoxon Rank Sum test); and the area-under-the-curve (AUC) values in receiver-operating-characteristic (ROC) analysis also were not statistically significantly different between the groups for the differentiation of cancer from healthy subjects (S2 File). Therefore in the subsequent analyses we grouped all cancer patients together.

We used the AUC to determine the ability of CA 19–9 to distinguish the groups. The CA19-9 assays were best at separating the cancer from healthy control groups (AUCs equal to 0.78 with 95% CI (0.70,0.84) for Assay 1, and 0.76 (0.68, 0.83) for Assay 2) (Fig 1B). The assays also had good performance in separating the cancer from chronic pancreatitis groups (AUCs equal to 0.73 with 95% CI (0.65, 0.80) for Assay 1, and 0.77 (0.69, 0.83) for Assay 2) (Fig 1C), but they were not effective at separating the cancer from the benign biliary obstruction groups (AUCs equal to 0.56 with 95% CI (0.47, 0.67) for Assay 1, and 0.62 (0.45, 0.71) for Assay 2) (Fig 1D). There were no statistically significant differences in AUCs between Assays 1 and 2 in each of these comparisons.

To more directly relate these results to clinical practice, we examined the sensitivities and specificities of the CA19-9 assays at the typical cutoff used in practice, 37 U/mL, and at cutoffs that gave maximum sums of sensitivity and specificity (Table 2). At the 37 U/mL cutoff, the average of the sensitivity plus specificity ranged from 70 to 74 for the discrimination of cancer from the healthy and chronic pancreatitis control groups. For both assays, a lower threshold greatly increased sensitivity with a lesser decrease in specificity, resulting in a statistically significant improvement in average sensitivity and specificity (Table 2).

**Table 2:** Marker performance at selected cutoffs.

Markers	AUC(95%CI)	Marker Cutoff (U/mL)		Sensitivity (95%CI)	Specificity (95%CI)	(Sens+Spec)/2 (95%CI)
		Assay 1	Assay 2			
**All cancer vs. healthy subjects**						
Assay 1 –optimized cutoff	0.78 (0.70–0.84)	23.165		66.3 (54.1–77.6)	90.2 (82.0–98.4)	78.2 (73.3–84.3)
Assay 1 –clinical cutoff		37		54.1 (43.9–63.8)	95.1 (88.5–100.0)	74.6 (68.6–80.4)
Assay 2 –optimized cutoff	0.76 (0.68–0.83)		16.64	75.5 (57.6–83.7)	75.4 (67.2–92.7)	75.5 (70.1–82.1)
Assay 2 –clinical cutoff			37	53.1 (42.9–62.2)	88.5 (80.3–95.1)	70.8 (64.7–76.8)
AND–optimized cutoffs		22.00	14.05	65.3 (56.6–81.1)	91.8 (77.0–100.0)	78.6 (74.0–84.7)
OR–optimized cutoffs		23.40	170.85	67.3 (56.1–82.7)	90.2 (75.4–98.4)	78.8 (74.5–84.9)
AND–clinical cutoffs		37	37	45.9 (35.7–56.1)	95.1 (88.5–100.0)	70.5 (64.9–76.0)
OR–clinical cutoffs		37	37	61.2 (50.0–70.9)	88.5 (80.3–95.1)	74.9 (68.9–80.8)
**All cancer vs. chronic pancreatitis**						
Assay 1 –optimized cutoff	0.73 (0.65–0.80)	29.78		61.2 (43.9–75.5)	82.3 (71.0–96.8)	71.7 (67.3–79.6)
Assay 1 –clinical cutoff		37		54.1 (43.9–65.3)	87.1 (79.0–95.2)	70.6 (64.0–77.3)
Assay 2 –optimized cutoff	0.77 (0.69–0.83)		25.25	65.3 (54.1–78.6)	87.1 (74.2–96.8)	76.2 (70.6–82.4)
Assay 2 –clinical cutoff			37	53.1 (42.9–63.3)	91.9 (83.9–97.6)	72.5 (65.8–78.6)
AND–optimized cutoffs		0.0	25.66	65.3 (52.0–76.5)	87.1 (79.0–97.6)	76.2 (71.8–83.1)
OR–optimized cutoffs		42.00	25.66	68.4 (56.1–78.6)	85.5 (77.4–95.2)	76.9 (72.2–83.4)
AND–clinical cutoffs		37	37	45.9 (35.7–56.1)	95.2 (88.7–100.0)	70.5 (64.4–76.2)
OR–clinical cutoffs		37	37	61.2 (51.0–71.4)	83.9 (74.2–91.9)	72.5 (65.6–79.1)

### Patient-by-patient comparison of the CA 19–9 assays

**Fig 2:**
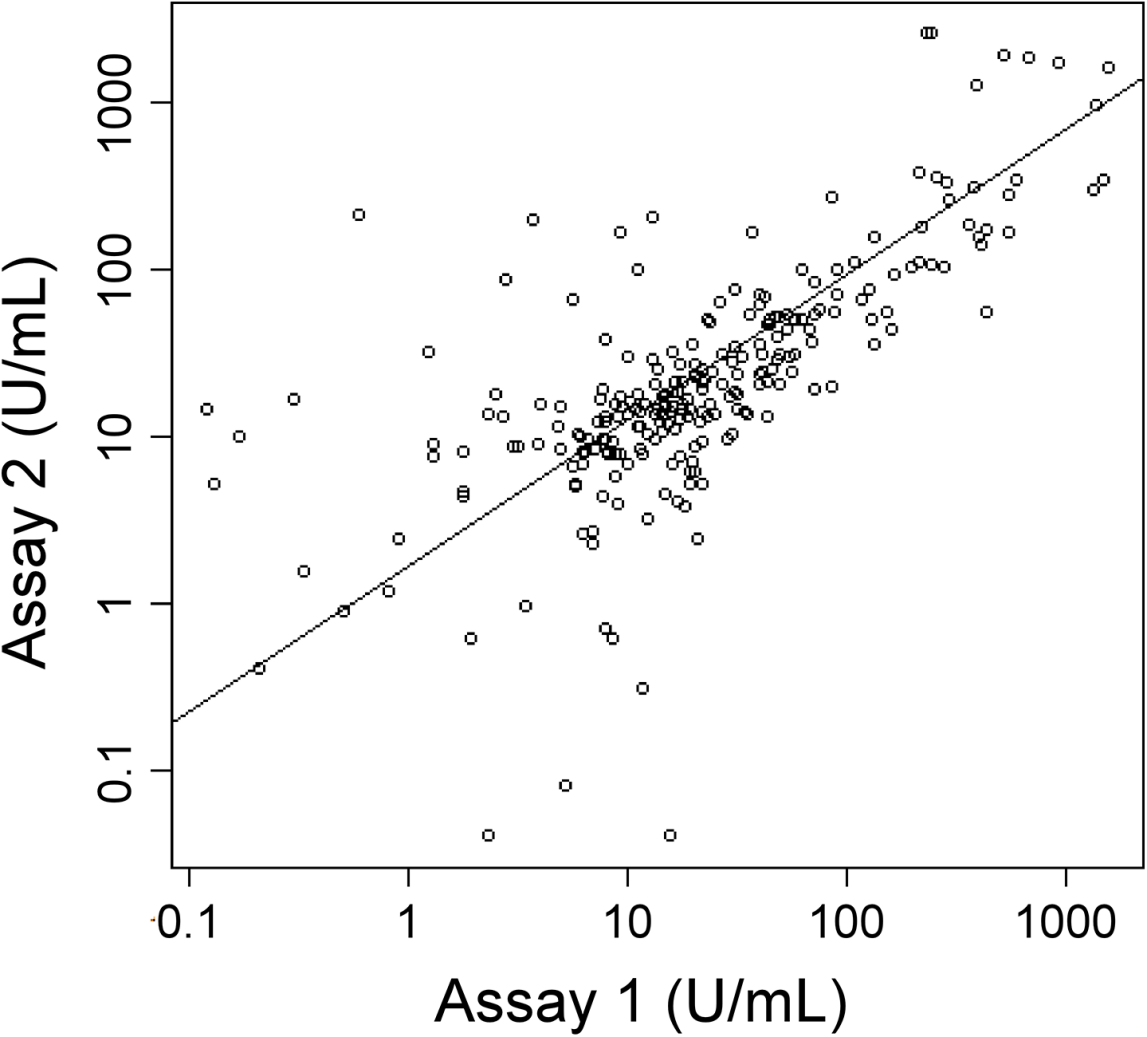
Correlation between Assay 1 and Assay 2. The log-transformed CA 19–9 values of all subjects from Assay 1 and Assay 2 were plotted with respect to each other. Each circle indicates an individual patient sample. The trendline is the linear-least squares best fit, and the Spearman’s rank correlation coefficient is 0.74.

The above analysis shows that CA 19–9 performance is similar between Assay 1 and Assay 2 when evaluated over all patients, but we also wanted to know how the two assays compared for individual patients. A direct comparison of the values obtained for each patient showed major differences for certain patients (Fig 2). The discrepancies between the assays were evenly distributed; in some cases Assay 1 was higher, and in other cases Assay 2 was higher.

At a practical level, discrepancies between the assays only matter in relation to the cutoffs used in clinical practice. Therefore we queried how often the status of a sample was different between Assay 1 and Assay 2 in relation to the cutoff (Table 3). Using 37 U/mL as the cutoff, 8 of the 53 cancer patients (15%) elevated in Assay 1 were low in Assay 2, and 7 of the 52 cancer patients (14%) elevated in Assay 2 were low in Assay 1. Seven of the 128 control subjects (5%) not elevated in Assay 1 were elevated in Assay 2, and 9 of the 130 controls (7%) not elevated in Assay 2 were elevated in Assay 1. Discrepancies also were present using a 100 U/mL cutoff. Thus, although the two assays give generally the same overall performance, differences were apparent for individual patients. Each assay occasionally gave false negative or false positive results as compared to the other.

**Table 3:** Deviations between the assays at specific cutoffs.

	Cancer Patients				Control Subjects		
		Assay 2				Assay 2	
	37 U/mL	Low	High		37 U/mL	Low	High
Assay 1	Low	38	7	Assay 1	Low	121	7
	High	8	45		High	9	17
	Cancer Patients				Control Subjects		
		Assay 2				Assay 2	
	100 U/mL	Low	High		100 U/mL	Low	High
Assay 1	Low	68	2	Assay 1	Low	139	5
	High	4	24		High	4	6

### Testing the CA 19–9 assays in combination

We evaluated whether a combination of the two assays gave better results than either assay alone. This concept is based on the premise that if the antibodies in each assay optimally detect distinct subsets of patients, a greater percentage of patients would be detected if the assays were combined. We used logistic regression to derive linear combinations of the log-transformed values of the two assays, and evaluated the AUCs for separating cancer from each of the control groups based on the combined score. Combining the two assays did not show significant improvement for distinguishing cancer from the healthy or the chronic pancreatitis groups (Fig 1 and Table 2). In addition, using simple combination rules based on AND or OR operators, at either optimized cutoffs or the standard 37 U/mL cutoff, there was minimal improvement in average sensitivity and specificity relative to the individual assays (Table 2). Therefore, although the two assays potentially identify non-overlapping sets of patients (as suggested by the lack of perfect correlation in Fig 2), they do not contribute complementary, cancer-specific information.

### Specificity differences between the CA 19–9 antibodies

More information about the origin of the differences between the assays could be helpful to optimize the detection of cancer patients while minimizing detection of control subjects. The antigen nominally recognized by CA 19–9 antibodies is a glycan called sialyl Lewis A [16], with the sequence Siaα2,3Galβ1,3(Fucα1,4)GlcNAc, where Sia is sialic acid, Gal is galactose, Fuc is fucose, and GlcNAc is N-acetylglucosamine. Sialyl Lewis A is a member of the Lewis blood group system of glycans on red blood cells and a variety of glycoproteins and glycolipids [22, 28]. Although sialyl Lewis A is the main glycan bound by CA 19–9 antibodies, other glycans may be bound [19, 29]. A powerful tool for getting more information about what other glycans an antibody binds is the glycan array [24, 30], which enables measurements of the binding of a protein to many different glycans in parallel. We therefore obtained glycan array data for the antibodies used in Assays 1 and 2.

**Fig 3:**
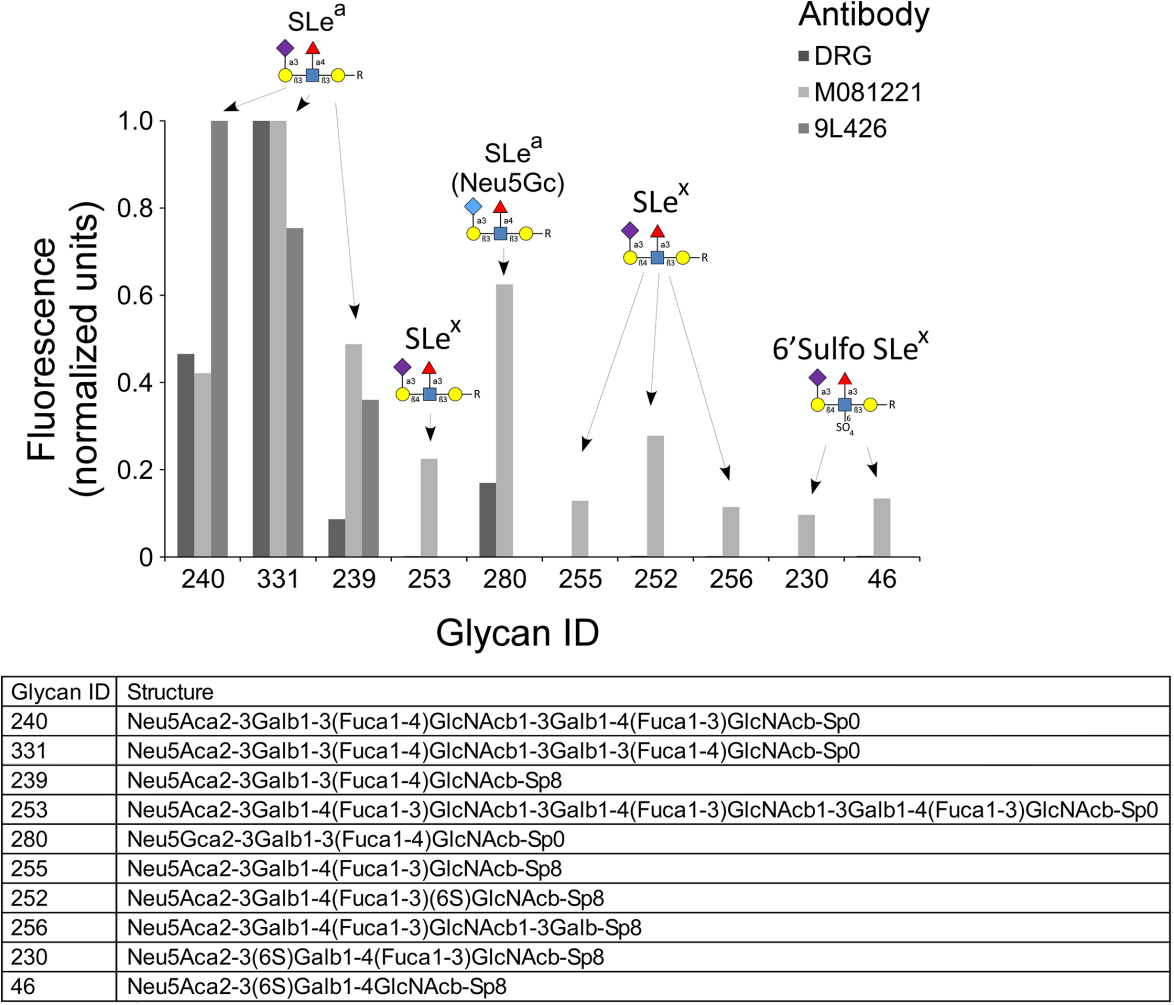
Glycan array analysis of three CA 19–9 antibodies. Each antibody was incubated at 2 μg/mL on a glycan array containing 610 distinct glycans (glycan array version 5.1 from the Consortium for Functional Glycomics). The graph includes the glycans showing the highest levels of binding by any of the antibodies. For each antibody, we normalized the raw fluorescence values to set the highest value to 1. The list specifies the glycans included in the plot, and the labels above the columns indicate the primary motif in each glycan. DRG is the Assay 1 antibody, 9L426 is the Assay 2 antibody, and M081221 is another anti-sialyl Lewis A antibody included for comparison.

**Fig 4:**
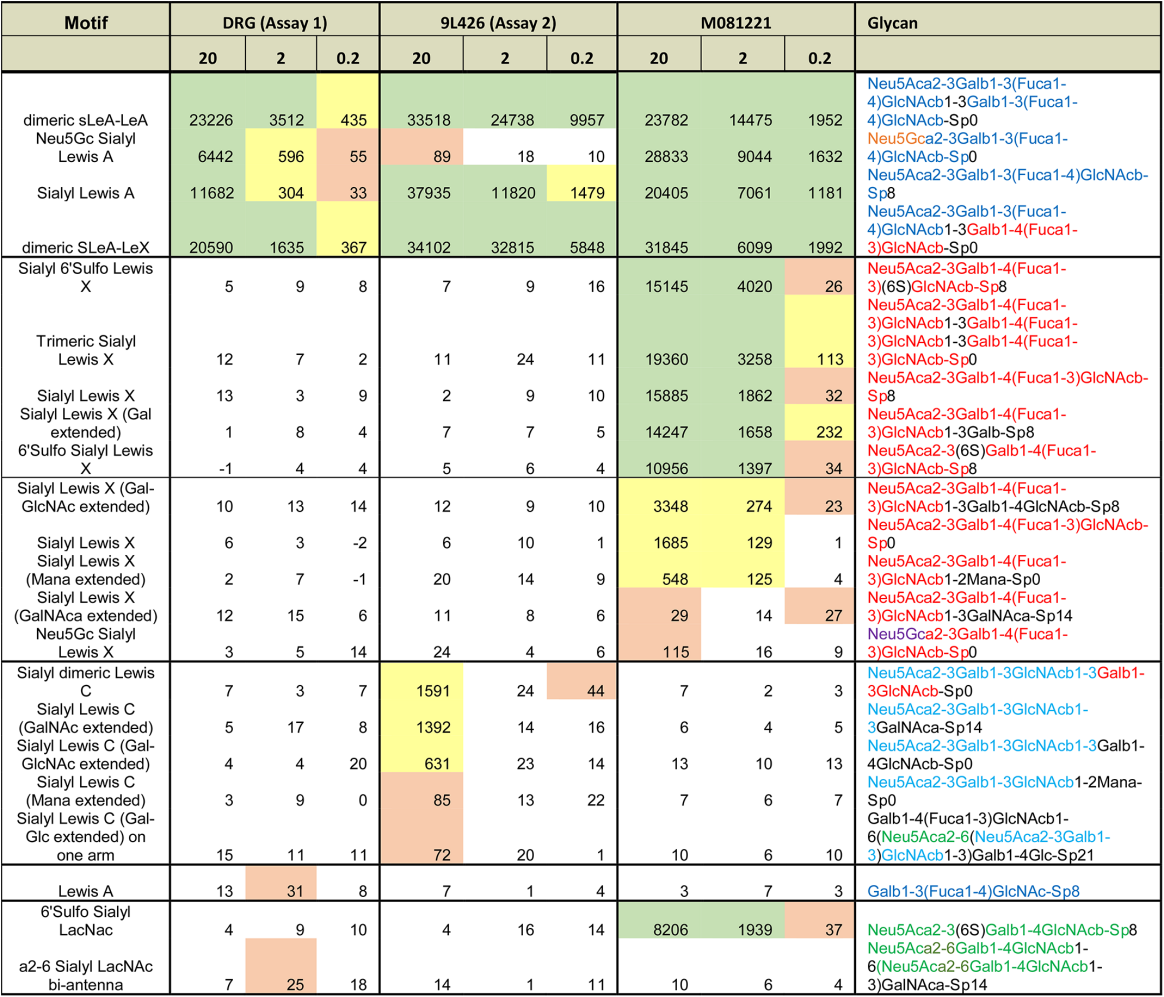
Glycan motifs recognized by three CA 19–9 antibodies. For each antibody, the binding intensity to the indicated glycans, in relative fluorescence units, is given at three antibody concentrations (in μg/mL). Green indicates strong binding, yellow is moderate binding, and pink is weak binding.

Both antibodies bound the canonical CA 19–9 antigen—sialyl Lewis A—but with differences (Fig 3 and Fig 4). The antibody from Assay 2 (9L426) bound more strongly than the Assay 1 antibody to dimeric sLeA-LeA, as evidenced by the better signal at the low concentration of 0.2 μg/mL, but it showed no binding to the sialic acid variant Neu5Gc. It also bound to sialyl Lewis C (non-fucosylated sialyl Lewis A) at the relatively high concentration of 20 μg/mL. In contrast, the Assay 1 antibody (only the detection antibody was available from the commercial kit) showed greater binding to Neu5Gc but no additional binding to sLeC. A third antibody (clone M081221, Fitzgerald, Acton, MA), not used in Assay 1 or 2 but included for comparison, bound other glycans in addition to those displaying the canonical CA 19–9 antigen. This antibody cross-reacted with glycans containing sialyl Lewis X, an isomer of sialyl Lewis A in which the attachment of the fucose and galactose to the core structure is switched. Thus the CA 19–9 antibodies have overlapping but distinct specificities, including binding to some structures beyond the canonical CA 19–9 antigen.

## Discussion

The reference set presented here is intended to facilitate reliable and rapid assessment of the performance of candidate biomarkers for the detection of pancreatic cancer. The large cohort of resectable pancreatic cancer, the furnishing of samples from multiple institutions using a standard procedure, and the inclusion of chronic pancreatic and benign biliary obstruction control groups establish the value of the set for validating assays. A first step in using the reference set was to characterize the performance of the CA 19–9 assay, in order to provide a benchmark for subsequent candidate biomarkers, to give a definitive evaluation of CA 19–9 in resectable pancreatic cancer, and to enable assessments of the combined use of candidate biomarkers and CA19-9. The use of two assays for CA 19–9 provided additional details about the biomarker, namely that subtle differences in specificity can affect results for individual patients but not necessarily for overall statistics.

The cohort of stage I-II pancreatic cancer patients in the reference set is substantially larger than what is typically available for pancreatic cancer biomarker studies, so the results presented here likely provide the most reliable information currently available about CA 19–9 in stage I-II pancreatic cancer. The main reason for the lack of large studies on early-stage pancreatic cancer has been the difficulty in obtaining the samples. Typical biomarker studies in pancreatic cancer include a small minority of stage I/II patients, such as a study in which 10 of the 67 pancreatic cancer patients had stage I/II disease [31]. Many studies do not specify the stage of the patients but likely involve mostly late stage patients because samples from late-stage patients are more readily available. The performance of CA 19–9 observed here using only stage I-II cases is comparable to that of previous studies using a mix of stages [17]. A review of 22 different studies of CA 19–9 for the diagnosis of pancreatic cancer reported 79% sensitivity and 82% specificity, averaged over all studies [17]. Previous studies found increases in average CA 19–9 levels with tumor stage and tumor size [21, 32–34]. The sensitivities were 40%, 68%, and 89% for T1 (tumor < 2 cm), T2 (< 2 cm and > 4 cm), T3 (> 4 cm), respectively [32]. Our determination of sensitivity at 54% for stage I-II cancer at the clinical cutoff of 37 U/mL (Table 2) is consistent with these findings. Taken together, our results show that many small, organ-confined cancers secrete appreciable levels of CA 19–9 antigen into the blood, and that a portion of pancreatic cancers does not secrete CA 19–9 even at later stages. The discovery of markers that are complementary to CA 19–9 is needed to detect the full spectrum of pancreatic cancers.

Samples from chronic pancreatitis patients were included because of known difficulties in distinguishing this condition from pancreatic cancer. Chronic pancreatitis patients did not show elevations in CA 19–9 relative to the healthy control subjects. Other studies have found elevations above 37 U/mL in 18–21% of patients with chronic pancreatitis [32, 33]. The variance between the results of our study and those of some previous studies may be due to differences in the selection of the chronic pancreatitis patients, for example due to greater care in this study to select confirmed chronic pancreatitis patients in a quiescent state. In any case, the observations of higher CA 19–9 levels in cancer relative to chronic pancreatitis have led some to suggest the use of higher thresholds (>100 U/mL) for the differential diagnosis of pancreatic cancer from chronic pancreatitis [31, 32, 34, 35]. Our study confirmed that elevations can be confirmatory for stage I-II pancreatic cancer with a sensitivity of ~70% at thresholds that give near perfect specificity. Therefore CA 19–9 can provide very reliable confirmatory information, but only for the subset of patients with moderate and greater elevations.

We included samples from patients with benign biliary obstruction—defined as biliary obstruction in the absence of pancreatitis or pancreatic cancer—to gather more information about the sources of biomarker elevation, since masses in the pancreas arising from cancer or pancreatitis can cause biliary obstruction. The present study agreed with previous studies showing that biliary obstruction can cause elevations in CA 19–9 nearly equivalent to those of early-stage pancreatic cancer [36, 37]. This fact has led to speculation that the elevations of CA 19–9 in all pancreatic diseases are simply a secondary effect from biliary obstruction, rather than due to secretions from the pancreatic parenchyma. Several lines of evidence suggest otherwise. Pancreatic cancer patients without jaundice often have CA 19–9 elevations; some cancer patients continue to have elevated CA 19–9 even after the relief of biliary obstruction; and immunohistochemical analysis of CA 19–9 in pancreatic cancer show thick perfusion of the cancerous tissue with CA 19–9, showing direct secretion from the cancer cells. Therefore, in cases where biliary obstruction is not present or has been resolved, CA 19–9 elevations in the serum likely result directly from cancer cell secretions. Future studies using the reference set should benefit from a similar analysis of benign biliary obstruction cases, especially considering that jaundice is a known source of non-specific elevation for some protein biomarkers [38].

The comparison of CA 19–9 assays has implications for interpretation of the values. The two assays agreed very well in their summary statistics and overall trends, but the values did not always agree for individual patients (Fig 2), in some cases leading to differences in status relative to the 37 U/mL or 100 U/mL cutoffs (Table 3). We conclude that the interpretation and comparison of CA 19–9 values should always take into account the assay used. This finding is consistent with previous research showing divergence between CA 19–9 assays [27] and a previous study linking that divergence to the glycan-binding characteristics of the antibodies [19].

A previous study examined the binding of 20 different monoclonal antibodies against sialyl Lewis A to 9 different glycan structures [39]. The study agreed with our results in finding variation between the antibodies (none of the 20 were in our study), but it did not include several important glycans similar to sialyl Lewis A, such as sulfated glycans, sialyl Lewis C, and Lewis Y, and it provided no information about the effects of branching or extension. The glycan arrays used in the present study contained over 600 glycans, including glycans similar to sialyl Lewis A and unrelated glycans that serve as negative controls. Therefore we have a higher level of detail in the analysis of the specificities of the antibodies, which can serve to better interpret results obtained using the antibodies.

Other factors in addition to specificity differences could contribute to differences between the assays, such as precision and interference from heterophile antibodies. It is likely that such factors contributed to discrepancies, but there is evidence for a major contribution from specificity differences. The differences between assays for selected samples shown by the scatter plot of Fig 2 are larger than the imprecision in the assays, and because all antibodies were mouse monoclonals, heterophile interference likely would be similar between the assays. Future experiments also could delve into this question more deeply. One could characterize the glycans of samples that show major differences between the assays to determine whether particular glycan motifs were detected preferentially by one assay relative to the other.

In summary, we describe a resource for pancreatic cancer biomarker development and the use of this resource to advance our knowledge about the CA 19–9 test. The reference set has several features that make it ideal for biomarker testing, including a large cohort of resectable cancers, samples from multiple institutions, and the inclusion of the key control groups. The value of using a common sample set was demonstrated by the use of two assays for CA 19–9, as they agreed in their summary statistics but were divergent for individual patients. The reference set promises to be a valuable resource for biomarker validation and comparison, and future studies of candidate biomarkers in the reference set could be compared and integrated with the present results.

## Supporting Information

S1 File Supplementary methods. CA 19–9 Assays; Data Processing and Assay Characteristics
(DOCX)

S2 File CA19-9 data from Assays 1 and 2. (XLSX)

S3 File Supplementary tables and figures. (DOCX)

## References

[1] Li D, Abbruzzese JL. New strategies in pancreatic cancer: emerging epidemiologic and therapeutic concepts. Clin Cancer Res. 2010;16(17):4313–8. Epub 2010/07/22. 1078-0432.CCR-09-1942 [pii] 10.1158/1078-0432.CCR-09-1942 20647474 PMC2932833

[2] Asuthkar S, Rao JS, Gondi CS. Drugs in preclinical and early-stage clinical development for pancreatic cancer. Expert Opin Investig Drugs. 2012;21(2):143–52. Epub 2012/01/06. 10.1517/13543784.2012.651124 . 22217246

[3] Iacobuzio-Donahue CA, Velculescu VE, Wolfgang CL, Hruban RH. Genetic basis of pancreas cancer development and progression: insights from whole-exome and whole-genome sequencing. Clinical Cancer Research. 2012;18(16):4257–65. Epub 2012/08/17. 10.1158/1078-0432.CCR-12-0315 22896692 PMC3422771

[4] Olive KP, Jacobetz MA, Davidson CJ, Gopinathan A, McIntyre D, Honess D, et al Inhibition of Hedgehog signaling enhances delivery of chemotherapy in a mouse model of pancreatic cancer. Science (New York, NY. 2009;324(5933):1457–61. Epub 2009/05/23. 10.1126/science.1171362 19460966 PMC2998180

[5] Neesse A, Michl P, Frese KK, Feig C, Cook N, Jacobetz MA, et al Stromal biology and therapy in pancreatic cancer. Gut. 2011;60(6):861–8. Epub 2010/10/23. 10.1136/gut.2010.226092 . 20966025

[6] Von Hoff DD, Ervin T, Arena FP, Chiorean EG, Infante J, Moore M, et al Increased survival in pancreatic cancer with nab-paclitaxel plus gemcitabine. The New England journal of medicine. 2013;369(18):1691–703. Epub 2013/10/18. 10.1056/NEJMoa1304369 . 24131140 PMC4631139

[7] Whatcott C, Han H, Posner RG, Von Hoff DD. Tumor-stromal interactions in pancreatic cancer. Crit Rev Oncog. 2013;18(1–2):135–51. Epub 2012/12/15. 23237556 10.1615/critrevoncog.v18.i1-2.80 PMC3632415

[8] Rhim AD, Mirek ET, Aiello NM, Maitra A, Bailey JM, McAllister F, et al EMT and Dissemination Precede Pancreatic Tumor Formation. Cell. 2012;148(1–2):349–61. Epub 2012/01/24. S0092-8674(11)01369-9 [pii] 10.1016/j.cell.2011.11.025 . 22265420 PMC3266542

[9] Ludwig JA, Weinstein JN. Biomarkers in cancer staging, prognosis and treatment selection. Nat Rev Cancer. 2005;5(11):845–56. Epub 2005/10/22. nrc1739 [pii] 10.1038/nrc1739 . 16239904

[10] Ransohoff DF. Bias as a threat to the validity of cancer molecular-marker research. Nat Rev Cancer. 2005;5(2):142–9. . 15685197 10.1038/nrc1550

[11] Ransohoff DF. How to improve reliability and efficiency of research about molecular markers: roles of phases, guidelines, and study design. J Clin Epidemiol. 2007;60(12):1205–19. Epub 2007/11/14. S0895-4356(07)00283-1 [pii] 10.1016/j.jclinepi.2007.04.020 . 17998073

[12] Feng Z, Kagan J, Pepe M, Thornquist M, Ann Rinaudo J, Dahlgren J, et al The Early Detection Research Network's Specimen reference sets: paving the way for rapid evaluation of potential biomarkers. Clinical chemistry. 2013;59(1):68–74. Epub 2012/11/30. 10.1373/clinchem.2012.185140 23193062 PMC3652317

[13] O'Brien DP, Sandanayake NS, Jenkinson C, Gentry-Maharaj A, Apostolidou S, Fourkala EO, et al Serum CA19-9 is significantly upregulated up to 2 years before diagnosis with pancreatic cancer: implications for early disease detection. Clin Cancer Res. 2015;21(3):622–31. Epub 2014/06/19. 10.1158/1078-0432.ccr-14-0365 24938522 PMC4181906

[14] Menon U, Gentry-Maharaj A, Ryan A, Sharma A, Burnell M, Hallett R, et al Recruitment to multicentre trials–-lessons from UKCTOCS: descriptive study. BMJ (Clinical research ed). 2008;337:a2079 Epub 2008/11/15. 10.1136/bmj.a2079 19008269 PMC2583394

[15] Herlyn M, Steplewski Z, Herlyn D, Koprowski H. Colorectal carcinoma-specific antigen: detection by means of monoclonal antibodies. Proceedings of the National Academy of Sciences of the United States of America. 1979;76(3):1438–42. Epub 1979/03/01. 286328 10.1073/pnas.76.3.1438 PMC383267

[16] Magnani JL, Nilsson B, Brockhaus M, Zopf D, Steplewski Z, Koprowski H, et al A monoclonal antibody-defined antigen associated with gastrointestinal cancer is a ganglioside containing sialylated lacto-N-fucopentaose II. The Journal of biological chemistry. 1982;257(23):14365–9. Epub 1982/12/10. . 7142214

[17] Goonetilleke KS, Siriwardena AK. Systematic review of carbohydrate antigen (CA 19–9) as a biochemical marker in the diagnosis of pancreatic cancer. Eur J Surg Oncol. 2007;33(3):266–70. Epub 2006/11/14. S0748-7983(06)00376-3 [pii] 10.1016/j.ejso.2006.10.004 . 17097848

[18] Nishihara S, Yazawa S, Iwasaki H, Nakazato M, Kudo T, Ando T, et al Alpha (1,3/1,4)fucosyltransferase (FucT-III) gene is inactivated by a single amino acid substitution in Lewis histo-blood type negative individuals. Biochemical and biophysical research communications. 1993;196(2):624–31. Epub 1993/10/29. 10.1006/bbrc.1993.2295 . 8240337

[19] Partyka K, Maupin KA, Brand RE, Haab BB. Diverse monoclonal antibodies against the CA 19–9 antigen show variation in binding specificity with consequences for clinical interpretation. Proteomics. 2012;12:2212–20. Epub 2012/05/25. 10.1002/pmic.201100676 . 22623153 PMC3711024

[20] Balasenthil S, Chen N, Lott ST, Chen J, Carter J, Grizzle WE, et al A migration signature and plasma biomarker panel for pancreatic adenocarcinoma. Cancer Prev Res (Phila). 2011;4(1):137–49. Epub 2010/11/13. 10.1158/1940-6207.CAPR-10-0025 21071578 PMC3635082

[21] Yue T, Maupin KA, Fallon B, Li L, Partyka K, Anderson MA, et al Enhanced discrimination of malignant from benign pancreatic disease by measuring the CA 19–9 antigen on specific protein carriers. PLoS ONE. 2011;6(12):e29180 Epub 2012/01/06. 10.1371/journal.pone.0029180 PONE-D-10-06087 [pii]. 22220206 PMC3248411

[22] Yue T, Partyka K, Maupin KA, Hurley M, Andrews P, Kaul K, et al Identification of blood-protein carriers of the CA 19–9 antigen and characterization of prevalence in pancreatic diseases. Proteomics. 2011;11(18):3665–74. Epub 2011/07/14. 10.1002/pmic.201000827 . 21751362 PMC3517053

[23] Pepe MS. The statistical evaluation of medical tests for classification and prediction Oxford, UK: Oxford University Press; 2003.

[24] Blixt O, Head S, Mondala T, Scanlan C, Huflejt ME, Alvarez R, et al Printed covalent glycan array for ligand profiling of diverse glycan binding proteins. Proceedings of the National Academy of Sciences of the United States of America. 2004;101(49):17033–8. . 15563589 10.1073/pnas.0407902101 PMC534418

[25] Kletter D, Cao Z, Bern M, Haab B. Determining Lectin Specificity from Glycan Array Data using Motif Segregation and GlycoSearch Software. Current Protocols in Chemical Biology. 2013;5:1–13. 10.1002/9780470559277.ch130028 23839995 PMC4041489

[26] Stern P, Friedecky B, Bartos V, Bezdickova D, Vavrova J, Uhrova J, et al Comparison of different immunoassays for CA 19–9. Clin Chem Lab Med. 2001;39(12):1278–82. Epub 2002/01/19. 10.1515/cclm.2001.205 . 11798090

[27] Passerini R, Riggio D, Salvatici M, Zorzino L, Radice D, Sandri MT. Interchangeability of measurements of CA 19–9 in serum with four frequently used assays: an update. Clin Chem Lab Med. 2007;45(1):100–4. Epub 2007/01/25. 10.1515/CCLM.2007.003 . 17243924

[28] Magnani JL, Steplewski Z, Koprowski H, Ginsburg V. Identification of the gastrointestinal and pancreatic cancer-associated antigen detected by monoclonal antibody 19–9 in the sera of patients as a mucin. Cancer research. 1983;43(11):5489–92. . 6193872

[29] Manimala JC, Roach TA, Li Z, Gildersleeve JC. High-throughput carbohydrate microarray profiling of 27 antibodies demonstrates widespread specificity problems. Glycobiology. 2007;17(8):17C–23C. . 17483136 10.1093/glycob/cwm047

[30] Liu Y, Palma AS, Feizi T. Carbohydrate microarrays: key developments in glycobiology. Biol Chem. 2009;390(7):647–56. Epub 2009/05/12. 10.1515/BC.2009.071 . 19426131

[31] Andicoechea A, Vizoso F, Alexandre E, Martinez A, Cruz Diez M, Riera L, et al Comparative study of carbohydrate antigen 195 and carcinoembryonic antigen for the diagnosis of pancreatic carcinoma. World J Surg. 1999;23(3):227–31; discussion 31–2. Epub 1999/02/06. . 9933690 10.1007/pl00013182

[32] Kobayashi T, Kawa S, Tokoo M, Oguchi H, Kiyosawa K, Furuta S, et al Comparative study of CA–50 (time-resolved fluoroimmunoassay), Span–1, and CA19-9 in the diagnosis of pancreatic cancer. Scand J Gastroenterol. 1991;26(7):787–97. Epub 1991/07/01. . 1896821 10.3109/00365529108998600

[33] Satake K, Takeuchi T. Comparison of CA19-9 with other tumor markers in the diagnosis of cancer of the pancreas. Pancreas. 1994;9(6):720–4. Epub 1994/11/01. . 7846015 10.1097/00006676-199411000-00008

[34] Malesci A, Tommasini MA, Bonato C, Bocchia P, Bersani M, Zerbi A, et al Determination of CA 19–9 antigen in serum and pancreatic juice for differential diagnosis of pancreatic adenocarcinoma from chronic pancreatitis. Gastroenterology. 1987;92(1):60–7. Epub 1987/01/01. . 3465666 10.1016/0016-5085(87)90840-7

[35] Steinberg W. The clinical utility of the CA 19–9 tumor-associated antigen. The American journal of gastroenterology. 1990;85(4):350–5. . 2183589

[36] Ohshio G, Manabe T, Watanabe Y, Endo K, Kudo H, Suzuki T, et al Comparative studies of DU-PAN–2, carcinoembryonic antigen, and CA19-9 in the serum and bile of patients with pancreatic and biliary tract diseases: evaluation of the influence of obstructive jaundice. The American journal of gastroenterology. 1990;85(10):1370–6. Epub 1990/10/01. . 2220731

[37] Kim HJ, Kim MH, Myung SJ, Lim BC, Park ET, Yoo KS, et al A new strategy for the application of CA19-9 in the differentiation of pancreaticobiliary cancer: analysis using a receiver operating characteristic curve. The American journal of gastroenterology. 1999;94(7):1941–6. Epub 1999/07/16. 10.1111/j.1572-0241.1999.01234.x . 10406263

[38] Jenkinson C, Elliott V, Menon U, Apostolidou S, Fourkala OE, Gentry-Maharaj A, et al Evaluation in pre-diagnosis samples discounts ICAM–1 and TIMP–1 as biomarkers for earlier diagnosis of pancreatic cancer. J Proteomics. 2015;113:400–2. Epub 2014/10/16. 10.1016/j.jprot.2014.10.001 . 25316052

[39] Rye PD, Bovin NV, Vlasova EV, Molodyk AA, Baryshnikov A, Kreutz FT, et al Summary report on the ISOBM TD–6 workshop: analysis of 20 monoclonal antibodies against Sialyl Lewisa and related antigens. Montreux, Switzerland, September 19–24, 1997. Tumour Biol. 1998;19(5):390–420. Epub 1998/08/14. . 9701730 10.1159/000030032

