# Supplementary material for: Definitive Characterization of CA 19-9 in Resectable Pancreatic Cancer Using a Reference Set of Serum and Plasma Specimens
Source: PLoS One. 2015 Oct 2;10(10):e0139049. doi: 10.1371/journal.pone.0139049 (PMC4592020; doi:10.1371/journal.pone.0139049)
Supplement: S1 File — CA 19–9 Assays; Data Processing and Assay Characteristics (DOCX) [file pone.0139049.s001.docx]

Supplementary Methods, Haab et al., “Definitive characterization of CA 19-9 in early-stage pancreatic cancer using a reference set of serum and plasma specimens”

**CA 19-9 Assays**

We ran Assay 2 using a previously published protocol ([17](#_ENREF_17),[19](#_ENREF_19),[20](#_ENREF_20)), which is briefly described here. Forty-eight identical antibody arrays were printed onto glass microscope slides coated with ultra-thin nitrocellulose (PATH Slides, Grace BioLabs) using a contact printer (Aushon 2470, Aushon BioSystems). Six replicates of the capture antibody (Clone 9L426, US Biological, Salem, MA) were printed and randomized within each array. After printing, hydrophobic boarders were imprinted onto the slides (SlideImprinter, The Gel Company, San Francisco, CA) to segregate the arrays and allow for individual sample incubations on each array.

The arrays were blocked using 1% bovine serum albumin (BSA) in phosphate buffered saline (PBS) plus 0.5% Tween-20 for one hour at room temperature. The plasma samples were diluted two-fold into PBS containing 0.1% Tween-20, 0.1% Brij-35, an IgG blocking cocktail (200 μg/mL mouse and rabbit IgG and 100 μg/mL goat and sheep IgG (Jackson ImmunoResearch Inc.)) and protease inhibitor (Complete Mini EDTA-free Tablet, Roche Applied Science). After briefly washing the arrays in PBS/0.1% Tween-20, the samples were incubated on the arrays overnight at 4 °C. The arrays were washed in three changes of PBS/0.1% Tween-20 for three minutes each and dried by centrifugation (Eppendorf 5810R, rotor A-4-62, 1500 x g for three minutes), and a biotinylated CA 19-9 detection antibody (3 μg/mL in PBS with 0.1% BSA and 0.1% Tween-20) was incubated on the arrays for one hour at room temperature. The detection antibody was the same as the capture antibody except for the biotinylation, which we performed by reaction with N-hydroxy-succinimide-conjugated biotin (Cat. # 21336, Pierce Biotechnology). After washing and drying the arrays as above, phycoerythrin-conjugated streptavidin (Roche Applied Science) prepared at 2 μg/mL in PBS with 0.1% BSA and 0.1% Tween-20 was incubated for one hour at room temperature, followed by a final wash and dry. The arrays were scanned for fluorescence (LS Reloaded, Tecan) at 532 nm excitation, and the resulting images were analyzed using GenePix Pro 5.1 (Molecular Devices, Sunnyvale, CA).

Data Processing and Assay Characteristics

For both assays, we used a dilution series of a CA 19-9 standard to convert the signals to CA 19-9 Units (U). The standard for Assay 1 was supplied by the manufacturer, and the standard for Assay 2 was purchased separately (RayBiotech, cat# MD-16-0018P). The quantifiable response ranges were 0.12 to 600 U/mL for Assay 1 and 7.8 to 250 U/mL for Assay 2. After conversion to CA 19-9 Units, we averaged replicate measurements to arrive at the final values. For Assay 1, the assays were run in duplicate, with an average %error of 10% and a correlation coefficient of 0.96 between the duplicate values. For Assay 2, the assays were run in triplicate, with some samples repeated in additional triplicates to ensure the accuracy of the values. The average coefficient of variation over the replicate values was 24%.
