# Supplementary material for: Definitive Characterization of CA 19-9 in Resectable Pancreatic Cancer Using a Reference Set of Serum and Plasma Specimens
Source: PLoS One. 2015 Oct 2;10(10):e0139049. doi: 10.1371/journal.pone.0139049 (PMC4592020; doi:10.1371/journal.pone.0139049)
Supplement: S3 File — (DOCX) [file pone.0139049.s003.docx]

Supplementary Information, Haab et al., “Definitive characterization of CA 19-9 in resectable pancreatic cancer using a reference set of serum and plasma specimens”

**Table A. Distributions of each CA19-9 assay.** Assay 3 refers to the Abbott Architect CA 19-9 Immunoassay. The distributions are based on the 82 samples that were used in all three assays.

|  |  | Healthy | Chronic Pancreatitis | Benign Biliary Obstruction | Cancer |
| --- | --- | --- | --- | --- | --- |
| Median (IQR) | Assay 1 | 7.1 (1.7,15.6) | 12.4 (7.9,16.9) | 35.5 (14.6,55.1) | 45.3 (20.8,221.8) |
|  | Assay 2 | 13.6 (7.4,15.4) | 8.2 (4.8,20.2) | 24.8 (16,51.9) | 53.9 (19.7,125.3) |
|  | Assay 3 | 2.3 (2,7.3) | 5.5 (2.4,11.7) | 50.4 (7.7,107.6) | 147.9 (19,512.8) |
| P-value of WRS | 1 vs 2 | 0.208 | 0.33 | 0.796 | 0.811 |
|  | 1 vs 3 | 0.558 | 0.064 | 0.971 | 0.215 |
|  | 2 vs 3 | 0.002 | 0.406 | 0.912 | 0.172 |

IQR: Interquartile range (25% percentile to 75% percentile)

**Table B. Probabilities that the value of each assay is greater than 37 U/mL**. Assay 3 refers to the Abbott Architect CA 19-9 Immunoassay. The distributions are based on the 82 samples that were used in all three assays.

|  |  | Healthy | Chronic Pancreatitis | Benign Biliary Obstruction | Cancer |
| --- | --- | --- | --- | --- | --- |
| P(assay value > 37)x100% | Assay 1 | 5 (73.1,99.7) | 14.3 (62.6,96.2) | 50 (23.7,76.3) | 58.1 (25.1,60.7) |
|  | Assay 2 | 15 (61.1,96) | 4.8 (74.1,99.8) | 40 (27.4,86.3) | 58.1 (25.1,60.7) |
|  | Assay 3 | 5 (73.1,99.7) | 14.3 (62.6,96.2) | 60 (13.7,72.6) | 67.7 (17.3,51.5) |
| pvalue of McNemar's test | Assay 1 vs 2 | 0.48 | 0.48 | 1 | 1 |
|  | Assay 1 vs 3 | 1 | 1 | 1 | 0.45 |
|  | Assay 2 vs 3 | 0.48 | 0.48 | 0.48 | 0.45 |

**Table C. Distribution of CA19-9 for Assays 1 and 2.**

|  | Geometric mean (95% CI) | | | | |
| --- | --- | --- | --- | --- | --- |
| Assay 1  Assay 2 | Healthy  6.32 (3.87~10.31)  11.76 (8.58~16.13) | Chronic  10.06 (7.44~10.44)  10.08 (6.99~14.54) | Benign  25.99 (12.09~55.88)  20.86 (11.13~39.12) | Cancer  35.04 (22.53~54.51)  45.56 (32.11~64.65) |  |
| p-value (paired-t)*  p-value (Wilcoxon Signed Rank)* | 0.018  0.021 | 0.686  0.145 | 0.263  0.550 | 0.079  0.846 |  |

*p-value for comparing Assays 1 and 2

**Table D. P-values for comparing the distribution of CA19-9 between groups.** The values presented in lower and upper diagonals are based on Assay 1 and Assay 2, respectively. The p-values were calculated by the two-sample, Wilcoxon rank sum test.

|  | Healthy | Chronic pancreatitis | Benign biliary obstruction | Cancer |
| --- | --- | --- | --- | --- |
| Healthy |  | 0.533 (0.765) | 0.117 (0.006) | <0.0001(<0.0001) |
| Chronic pancreatitis | 0.084 (0.025) |  | 0.055 (0.004) | <0.0001 (<0.0001) |
| Benign biliary obstruction | 0.004 (<0.0001) | 0.058 (0.002) |  | 0.038 (0.049) |
| Cancer | <0.0001 (<0.0001) | 0.0002 (<0.0001) | 0.511(0.354) |  |


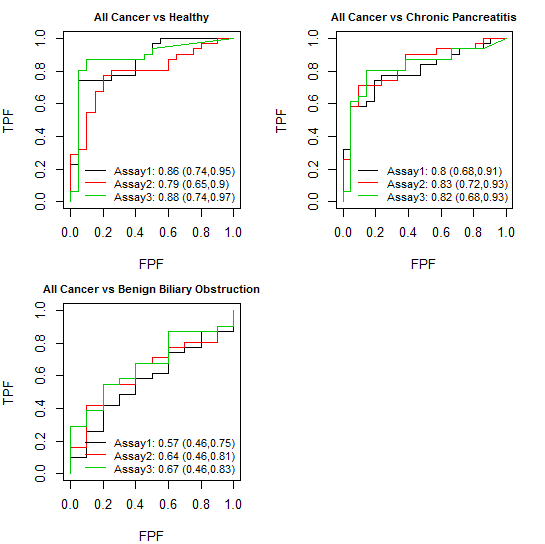


**Fig A. Receiver-operator characteristic (ROC) curves for the three assays.** Assay 3 refers to the Abbott Architect CA 19-9 Immunoassay, and the ROC curves are based on the 164 samples that were used in all three assays. The comparison groups are indicated at the top of each graph, and the legends indicate the area under the curve (AUC) for each assay, with the 95% confidence interval given in parentheses. No significant differences were observed between the assays for any of the comparisons.


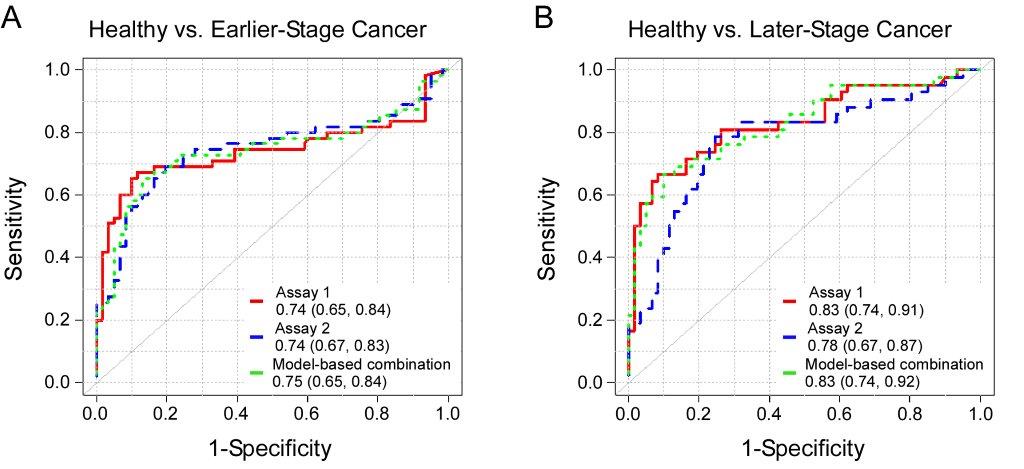


**Fig B. ROC curves for differentiating earlier and later stage cancer from healthy control subjects.** Earlier-stage cancer is defined as stages Ia, Ib, and IIa, and later-stage cancer is defined as stage IIb. We observed no differences in CA 19-9 levels between these stages.
